# Survival benefits with EPIC in addition to HIPEC for low grade appendiceal neoplasms with pseudomyxoma peritonei: a propensity score matched study

**DOI:** 10.1515/pp-2022-0205

**Published:** 2023-03-16

**Authors:** Raymond Hayler, Kathleen Lockhart, Shoma Barat, Ernest Cheng, Jasmine Mui, Raphael Shamavonian, Nima Ahmadi, Nayef Alzahrani, Winston Liauw, David Morris

**Affiliations:** Peritonectomy and Liver Cancer Unit, Department of Surgery, St George Hospital, NSW, Sydney, Australia; School of Clinical Medicine, St George & Sutherland Campus, UNSW Medicine & Health, Sydney, Australia; Faculty of Medicine and Health, Macquarie University, Sydney, Australia; College of Medicine, Imam Muhammad Ibn Saud Islamic University, Riyadh, Kingdom of Saudi Arabia; Department of Medical Oncology, St George Hospital, NSW, Sydney, Australia

**Keywords:** cytoreductive surgery, early postoperative intra-peritoneal chemotherapy, hyperthermic intraperitoneal chemotherapy, pseudomyxoma peritonei, survival analysis

## Abstract

**Objectives:**

Appendiceal cancer is a rare malignancy, occurring in roughly 1.2 per 100,000 per year. Low grade appendiceal neoplasams (LAMN) in particular can lead to pseudomyxoma peritonei (PMP), and respond poorly to systemic chemotherapy. Standard treatment includes cytoreduction surgery (CRS) with addition of heated intraoperative peritoneal chemotherapy (HIPEC). Several centres include early postoperative intraperitoneal chemotherapy (EPIC) however; the literature is mixed on the benefits. We aim to examine the benefits of additional EPIC through a propensity-matched analysis.

**Methods:**

Patients with LAMN with PMP who underwent cytoreductive surgery at St George hospital between 1996 and 2020 were included in this retrospective analysis. Propensity score matching was performed with the following used to identify matched controls; sex, age, American Society of Anesthesiologists (ASA) grade, peritoneal cancer index (PCI) and morbidity grade. Outcomes measured included length of stay and survival.

**Results:**

A total of 224 patients were identified of which 52 received HIPEC alone. Propensity matching was performed to identify 52 matched patients who received HIPEC + EPIC. Those receiving HIPEC + EPIC were younger at 54.3 vs. 58.4 years (p=0.044). There was a median survival benefit of 34.3 months for HIPEC + EPIC (127.3 vs. 93.0 months, p=0.02). Median length of stay was higher in those who received EPIC (25.0 vs. 23.5 days, p=0.028).

**Conclusions:**

In LAMN with PMP, the addition of EPIC to HIPEC with CRS improves overall survival in propensity score matched cases but results in prolonged hospitalisation. The use of EPIC should still be considered in selected patients.

## Introduction

Appendiceal cancer is a rare malignancy with a rising incidence, occurring in roughly 1.2 per 100,000 per year and making up 0.4% of all gastrointestinal malignancies [1], [2], [3]. Diagnosis is often made incidentally, either during surgery for other causes or histologically after removal for presumed appendicitis [3]. Classification is by histological subtyping, with mucinous appendiceal neoplasias including low and high grade appendiceal mucinous neoplasms (LAMN and HAMN) as well as adenocarcinoma [2, 4]. Prognosis varies depending on the subtype, stage, histological grading, mucin deposits outside of the right lower quadrant as well as the presence of intracellular mucin [5]. Pseudomyxoma peritonei (PMP) is a clinical syndrome of intraperitoneal accumulation of mucus due to mucinous neoplasia, most commonly arising from appendiceal neoplasia [4, 6]. PMP is split into low-and high-grade dependent on its histological features, also known as disseminated peritoneal adenomucinosis (DPAM) or peritoneal mucinous carcinomatosis (PMCA) for low and high-grade respectively [4].

LAMNs with PMP responds poorly to systemic chemotherapy and treatment historically consisted of debulking or drainage of mucinous disease alone, with a poor 10-year survival between of 32% [7]. Cytoreductive surgery and hyperthermic intraperitoneal chemotherapy (HIPEC) introduced by Sugarbaker [8] significantly improved the survival of these patients with studies showing a 10 year survival of 63% [7]. Early postoperative intraperitoneal chemotherapy (EPIC) was introduced over 30 years ago [9] and is an alternative and additional form of perioperative intraperitoneal chemotherapy. The use of EPIC compared to HIPEC declined due to concerns of increased morbidity and survival benefits of HIPEC over EPIC, as well as the increased resource utility required to deliver EPIC, often requiring patients to stay in intensive care unit (ICU). A systematic review performed showed mixed data, with nine of thirteen studies showing increased morbidity with EPIC (either alone or in combination with HIPEC) and those in direct comparison of HIPEC to HIPEC + EPIC, five out of seven studies showed survival benefit with EPIC addition [10].

In those that showed survival benefit [11, 12], there were criticisms proposed regarding selection bias in that patients were younger and less comorbid when selected for additional EPIC [10]. Ideally, a randomised control trial (RCT) should be performed to compare this and minimise bias. Given the significant resources involved in an RCT, techniques have been developed to mimic RCTs in non-randomised data. One of these strategies is propensity score matching, in which observed covariates are weighted using a logistic regression model to generate a score. This score is then matched between the control and treatment group, minimising bias in non-randomised samples to allow comparison of cohorts [13].

Using propensity score matching, we aim to examine overall survival (OS) for patients with low grade appendiceal cancer and peritoneal carcinomatosis being treated with peritonectomy and HIPEC, with and without the addition of EPIC between matched cases.

## Materials and methods

All patients with LAMN and DPAM who underwent cytoreductive surgery at St George hospital between January 1996 and August of 2020 were included in this retrospective analysis. Patients who were undergoing redo-peritonectomy were excluded.

### Patient selection

All patients in this analysis were originally assessed pre-operatively for fitness for surgery with workup including basic blood tests, dihydropyrimidine dehydrogenase (DPYD) levels and tumour markers. DPYD testing was performed as low levels can lead to severe toxicity from impaired metabolism of 5-fluorouracil, our agent used in EPIC treatment [14]. Computed topography imaging of chest, abdomen and pelvis were performed as standard, with magnetic resonance imaging of the liver and positron emission tomography used depending on the patient. All patients are discussed at a multidisciplinary meeting.

### Intraoperative care

Intraoperatively, all patients undergo cytoreductive surgery in accordance with the principals established by Sugarbaker [8]. The peritoneal cancer index (PCI) is calculated at the start of the operation to grade the volume of disease and completeness of cytoreduction (CC) recorded at the end of cytoreduction for macroscopic tumour left [15]. Post CRS, HIPEC is administered using the Coliseum (open) technique. The abdomen was initially primed with Dianeal PD4 (1.5%) Peritoneal Dialysis Solution, in later years switching to Plasmalyte 148, and heated to 41.5 degree Celsius, with chemotherapy infused when reaching this temperature. Agents used includes mitomycin C (12.5 mg/m^2^) for 90 min, oxaliplatin (350 mg/m^2^) for 30 min or cisplatin (100 mg/m^2^) for 90 min.

### Early postoperative intraperitoneal chemotherapy

For patients who underwent EPIC, our protocol first requires passing a leak test on day 1 where 1 L of 0.9% sodium chloride is infused through a peritoneal port or Tenckhoff catheter (placed intraoperatively) to assess for leakage around drain sites. Patients must have ability to tolerate the administered fluid, as well as maintain an intraabdominal pressure below 20 mmHg, adequate urine output (above 0.5 mL/kg/h), absence of major organ failure and no evidence of sepsis. If they pass this test, sump drains are clamped and 5-fluorouracil 650 mg/m^2^ is administered with 50 mEq sodium bicarbonate on days 2 up to day 6, releasing the infusion every 23 h to allow for the next cycle.

### Data collection

Demographics such as age, sex and American Society of Anaesthetists (ASA) score were recorded. Operative information such as PCI, CC score and HIPEC agent were collected. Length of stay in hospital and ICU were measured in days. Morbidity was defined using the Clavien–Dindo classification from Grades I to V, with grade III and IV complications defined as a major morbidity and grade V defined as a mortality [16].

All statistics were performed using IBM^®^ SPSS^®^ software Version 24. GraphPad Prism 7^®^ was used to create the Kaplan–Meier analysis for overall survival. Cox regression was used for proportional hazard ratio to measure survival probability over time The last follow up date for cases was included in the survival calculations and was marked as “lost to follow up” from that time point onwards. These cases were censored from the overall population for analysis of survival from that point onwards.

### Propensity score matching

A propensity score technique was used to match cases within groups using HIPEC as a control group to that of the HIPEC + EPIC group. Logistical regression with a 1:1 matching was implemented with a match tolerance set to 0.1. Sex, age, ASA, PCI, CC score, HIPEC agent and morbidity grade were used as the covariates.

A pre-and post-propensity matching analysis of variance was performed on the groups to determine distribution of clinical characteristics for determining differences between the groups before and after matching. Pearson’s χ^2^ test was used to compare frequency of major morbidity between groups. Variance analysis, univariate and multivariate analysis was performed on the pre- and post-propensity matched cohort using overall survival as the outcome to determine clinical characteristics impact on survival, where significance indicates effect on survival. Pearson correlation was then used in post-hoc analysis to report the strength of this relationship. The level of significance (p) was set to 0.05 with p values less than 0.05 considered as significantly significant.

## Results

There were 224 patients identified, 124 of which were female (55.4%). Of these, 52 (23.2%) underwent HIPEC alone and 172 (76.8%) HIPEC + EPIC. Table 1 shows the pre-propensity score matching clinical data and demographics between the HIPEC and HIPEC + EPIC group. There were significant differences between the HIPEC and HIPEC + EPIC groups in age (58.4 vs. 52.2 years, p=0.001), median CC score (1 vs. 0, p=0.014), choice of HIPEC agent (p<0.001) and ASA status (<0.001). There was no significant difference between the groups in either median grade of morbidity complication or frequency of major morbidity (p=0.495 and 0.492, respectively).

**Table 1:** Comparison of baseline characteristics pre-propensity score matching.

		Pre-propensity score matching			
Variables	Categories	All (n=224)	HIPEC (n=52)	HIPEC_EPIC (n=172)	p-Value
Sex	Male, n (%)	100 (44.6)	28 (53.8)	72 (41.9)	0.078
	Female	124 (55.4)	24 (46.2)	100 (58.1)	
Age, years	Years (mean [SD])	53.7 (13.4)	58.4 (13.5)	52.2 (13.1)	0.001
PCI	Mean (SD)	22.6 (10.9)	24.6 (12.7)	22.0 (10.2)	0.123
	0–10, n (%)	38 (17.0)	10 (19.2)	28 (16.3)	
	11–20	55 (24.6)	3 (5.8)	52 (30.2)	
	21–30	66 (29.5)	17 (32.7)	49 (28.5)	
	>30	65 (29.0)	22 (42.3)	43 (25.0)	
Morbidity grade	Median (range)	2.0 (0.0–5.0)	2.0 (0.0–5.0)	2.0 (0.0–4.0)	0.495
	Grade III/IV complication, n (%)	86 (38.4)	17 (32.7)	69 (40.1)	^a^0.492
CC score	Median (range)	0.0 (0.0–2.0)	1.0 (0.0–2.0)	0.0 (0.0–2.0)	0.014
	0–1, n (%)	220 (98.2)	49 (94.2)	171 (99.4)	
	2–3	4 (1.8)	3 (5.8)	1 (0.6)	
HIPEC agent	Mitomycin C, n (%)	215 (96.0)	44 (84.6)	171 (99.4)	<0.001
	Oxaliplatin	7 (3.1)	6 (11.5)	1 (0.6)	
	Cisplatin	1 (0.4)	1 (1.9)	0 (0.0)	
Protocol	HIPEC only, n (%)	52 (23.21)	–	–	
	HIPEC plus EPIC	172 (76.79)	–	–	
ASA status	Median (range)	3 (0–4)	3.0 (2–4)	3 (0–4)	<0.001
	0	11 (4.9)	0 (0.0)	11 (6.4)	
	1	15 (6.7)	0 (0.0)	15 (8.7)	
	2	70 (31.3)	12 (23.1)	58 (33.7)	
	3	113 (50.4)	34 (65.4)	79 (45.9)	
	4	15 (6.7)	6 (11.5)	9 (5.2)	
Tumour markers (mean [SD])	CA19.9	84.2 (221.2)	66.8 (147.9)	73.6 (198.7)	0.872
	CA125	32.0 (43.6)	31.3 (48.0)	37.7 (42.6)	0.389
	CEA	37.3 (96.1)	43.4 (126.9)	23.2 (50.4)	0.072
ICU stay, days	Median (range)	2.0 (1.0–80.0)	3.0 (1.0–68.0)	2.0 (1.0–80.0)	0.183
Total stay, days	Median (range)	23.0 (6.0–164.0)	23.5 (6.0–164.0)	25.0 (9.0–150.0)	0.114

HIPEC, hyperthermic intraperitoneal chemotherapy; EPIC, early postoperative intraperitoneal chemotherapy; CC, completeness of cytoreduction; ASA, American Society of Anaesthesiologists; ICU, intensive care unit; SD, standard deviation. ^a^Refers to Pearson’s χ^2^ test used for analysis of frequency for grade III/IV morbidity between groups.

Clinical characteristics and demographics between the post-propensity score matching HIPEC and HIPEC+EPIC groups are shown in Table 2. Similar to the pre-propensity matching, in comparing the HIPEC and HIPEC + EPIC groups there is significant difference noted in age (58.4 vs. 54.3, p=0.044) and choice of HIPEC agent (p=0.045). There are additional significant differences noted in CA19.9 (66.8 vs. 101.5, p<0.001), CEA (43.4 vs. 31.3, p=0.045) and total length of stay in days (23.5 vs. 25.0, p=0.028). There remained no significant difference in median morbidity grade or frequency of major morbidity.

**Table 2:** Comparison of baseline characteristics for patients’ post-propensity score matching.

		Pre-propensity score matching			
Variables	Categories	All (n=104)	HIPEC (n=52)	HIPEC_EPIC (n=52)	p-Value
Sex	Male, n (%)	58 (55.8)	28 (53.8)	30 (57.7)	0.865
	Female	46 (44.2)	24 (46.2)	22 (42.3)	
Age	Years [mean (SD)]	56.3 (12.6)	58.4 (13.5)	54.3 (11.3)	0.044
PCI	Mean (SD)	24.3 (11.5)	24.6 (12.7)	24.0 (10.2)	0.745
	0–10, n (%)	17 (16.4)	10 (19.2)	7 (13.5)	
	11–20	15 (14.4)	3 (5.8)	12 (23.1)	
	21–30	35 (33.7)	17 (32.7)	18 (34.6)	
	>30	37 (35.6)	22 (42.3)	15 (28.9)	
Morbidity grade	Median (range)	2.0 (0.0–5.0)	2.0 (0.0–5.0)	2.0 (0.0–4.0)	0.827
	Grade III/IV	38 (37.6)	17 (32.7)	21 (40.4)	^a^0.555
CC score	Median (range)	1.0 (0.0–2.0)	1.0 (0.0–2.0)	1.0 (0.0–2.0)	0.845
	0–1, n (%)	100 (96.2)	49 (94.2)	51 (98.1)	
	2–3	4 (3.9)	3 (5.8)	1 (1.9)	
HIPEC agent	Mitomycin C, n (%)	95 (91.3)	44 (84.6)	51 (98.1)	0.045
	Oxaliplatin	7 (06.7)	6 (11.5)	1 (1.9)	
	Cisplatin	1 (1.0)	1 (1.9)	0 (0.0)	
Protocol	HIPEC only, n (%)	52 (50.0)	–	–	
	HIPEC plus EPIC	52 (50.0)	–	–	
ASA status	Median (range)	3 (0–4)	3 (2–4)	3 (0–4)	0.234
	0	3 (2.9)	0 (0)	3 (5.8)	
	1	1 (1.0)	0 (0)	1 (1.9)	
	2	24 (23.1)	12 (23.1)	12 (23.1)	
	3	62 (59.6)	34 (65.4)	28 (53.9)	
	4	14 (13.5)	6 (11.5)	8 (15.4)	
Tumour markers (mean [median])	CA19.9	84.1 (221.2)	66.8 (147.9)	101.5 (276.3)	<0.001
	CA125	32.0 (43.6)	31.3 (48.0)	32.8 (39.3)	0.133
	CEA	37.3 (96.1)	43.4 (126.9)	31.3 (49.6)	0.045
ICU stay, days	Median (range)	3.0 (1.0–68.0)	3.0 (1.0–68.0)	2.0 (1.0–29.0)	0.128
Total stay, days	Median (range)	24.0 (6.0–164.0)	23.5 (6.0–164.0)	25.0 (9.0–94.0)	0.028

HIPEC, hyperthermic intraperitoneal chemotherapy; EPIC, early postoperative intraperitoneal chemotherapy; CC, completeness of cytoreduction; ASA, American society of anaesthesiologists; ICU, intensive care unit. ^a^Refers to Pearson’s χ^2^ test used for analysis of frequency for grade III/IV morbidity between groups.

Non-parametric testing of the clinical characteristics in relation to survival pre-propensity matching is shown in the left-hand side of Table 3. There was no statistical significance of impact on survival for sex, age, choice of HIPEC agent or length of stay in ICU. There was evidence of effect on survival noted with univariate analysis for PCI (p<0.001), morbidity grade (p=<0.001), CC score (p=0.003), ASA status (p=0.017), total length of stay (p=0.001) and tumour markers; CA19.9 (p<0.001), CA125 (p<0.007) and CEA (p=0.001). Multivariate analysis was statistically significant in the same categories, with the addition of choice of HIPEC agent (p=0.003) not present in univariate. Pearson correlation coefficients revealed positive correlation with all factors, with the strongest correlation seen in PCI at 0.376.

**Table 3:** Correlation and significance of variables on survivals in the HIPEC and HIPEC + EPIC groups for pre- and post-propensity matched cases.

	Pre-propensity matching			Post-propensity matching		
Variables	Pearson correlation	Univariate	Multivariate	Pearson correlation	Univariate	Multivariate
Sex	0.095	0.326	0.17	0.022	0.822	0.922
Age	0.018	0.559	0.83	−0.05	0.613	0.501
PCI	0.376	<0.001	<0.001	0.39	<0.001	<0.001
Morbidity grade	0.224	<0.001	0.001	0.323	0.001	0.001
CC score	0.231	0.003	<0.001	0.166	0.092	0.094
HIPEC agent	0.146	0.200	0.03	0.108	0.279	0.279
ASA status	0.21	0.017	0.002	0.232	0.018	0.02
CA125	0.164	0.007	0.015	0.147	0.137	0.146
CEA	0.254	0.001	<0.001	0.223	0.023	0.027
CA19.9	0.335	<0.001	<0.001	0.315	0.001	0.001
ICU stay, days	0.113	0.200	0.105	0.219	0.026	0.028
Total stay, days	0.239	0.001	<0.001	0.316	0.001	0.001

HIPEC, hyperthermic intraperitoneal chemotherapy; EPIC, early postoperative intraperitoneal chemotherapy; CC, completeness of cytoreduction; ASA, American society of anaesthesiologists; ICU, intensive care unit.

Post-matching analysis of variables in relation to survival is shown in the right-hand side of Table 3. The following impacted survival with univariate analysis for PCI (p<0.001), morbidity grade (p=0.001), ASA status (p=0.018), total length of stay (p=0.001) and tumour markers; CA19.9 (p=0.001) and CEA (p<0.023). CC score, choice of HIPEC agent and CA-125 no longer showed impact on survival. In addition, ICU length of stay had evidence of impact on survival (p=0.026). All factors with evidence of impact on survival were positive correlated, with the strongest relationship once again demonstrated by PCI score at 0.390. Age did not impact survival in pre- or post-propensity matching.

The addition of EPIC to HIPEC was associated with stronger survival in hazard ratios (HR) at all time points measured (Table 4). This effect increased at each interval, from a HR of 1.12 (95% CI 1.07–1.16) at six months, to a HR of 1.37 (95% CI 1.33–1.42) at 6 months.

**Table 4:** Hazard ratios for survival between HIPEC + EPIC compared to HIPEC alone across time.

Time, months	Hazard ratio for survival HIPEC + EPIC vs. HIPEC alone	95% confidence interval
6	1.12	1.07–1.16
12	1.16	1.11–1.20
36	1.19	1.15–1.24
60	1.37	1.33–1.42

HIPEC, hyperthermic intraperitoneal chemotherapy; EPIC, early postoperative intraperitoneal chemotherapy.

**Figure 1:**
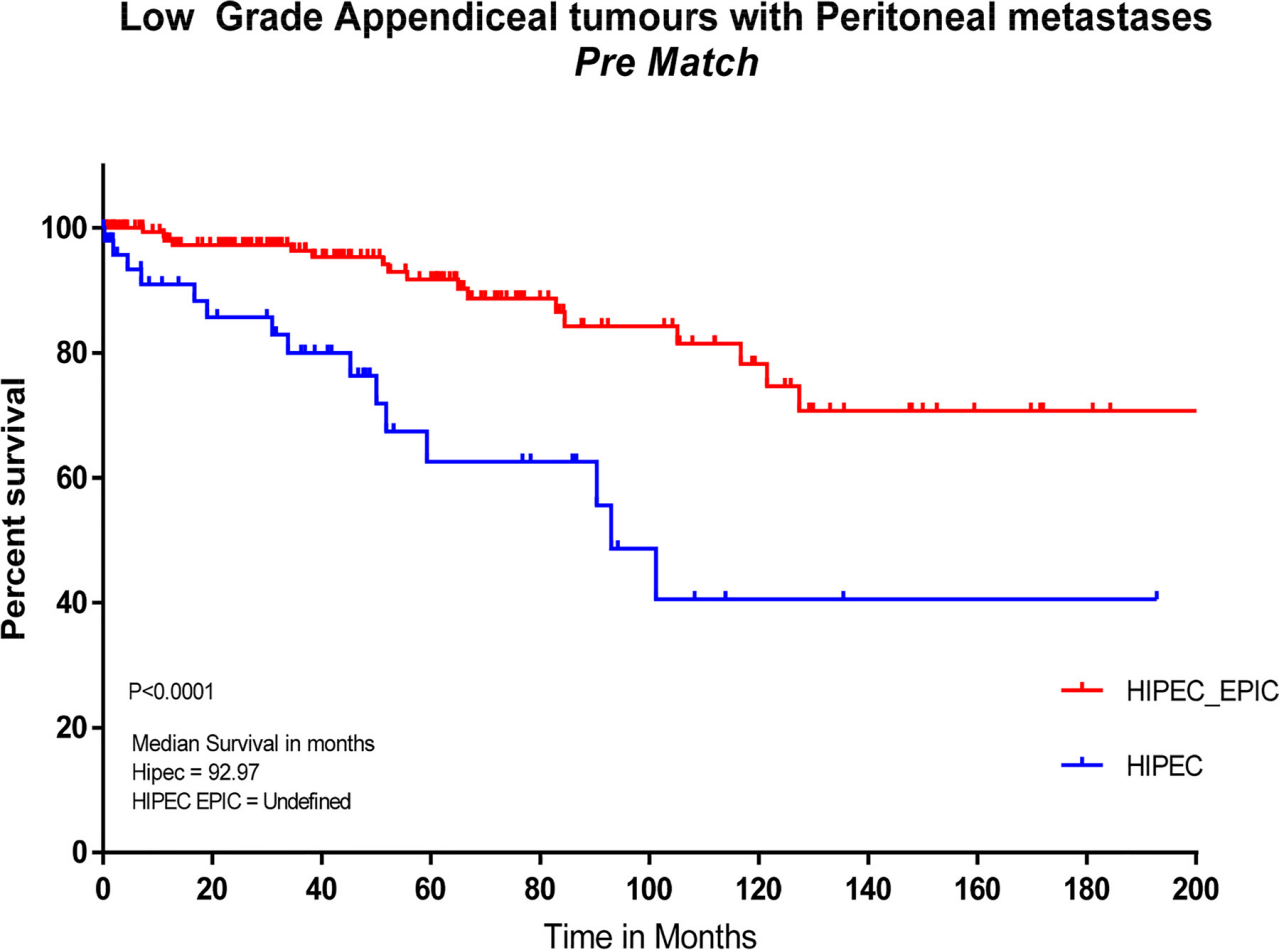
Kaplan-Meier curve of survival for patients treated with HIPEC or HIPEC + EPIC pre-propensity matching. HIPEC, hyperthermic intraperitoneal chemotherapy; EPIC, early postoperative intraperitoneal chemotherapy.

**Figure 2:**
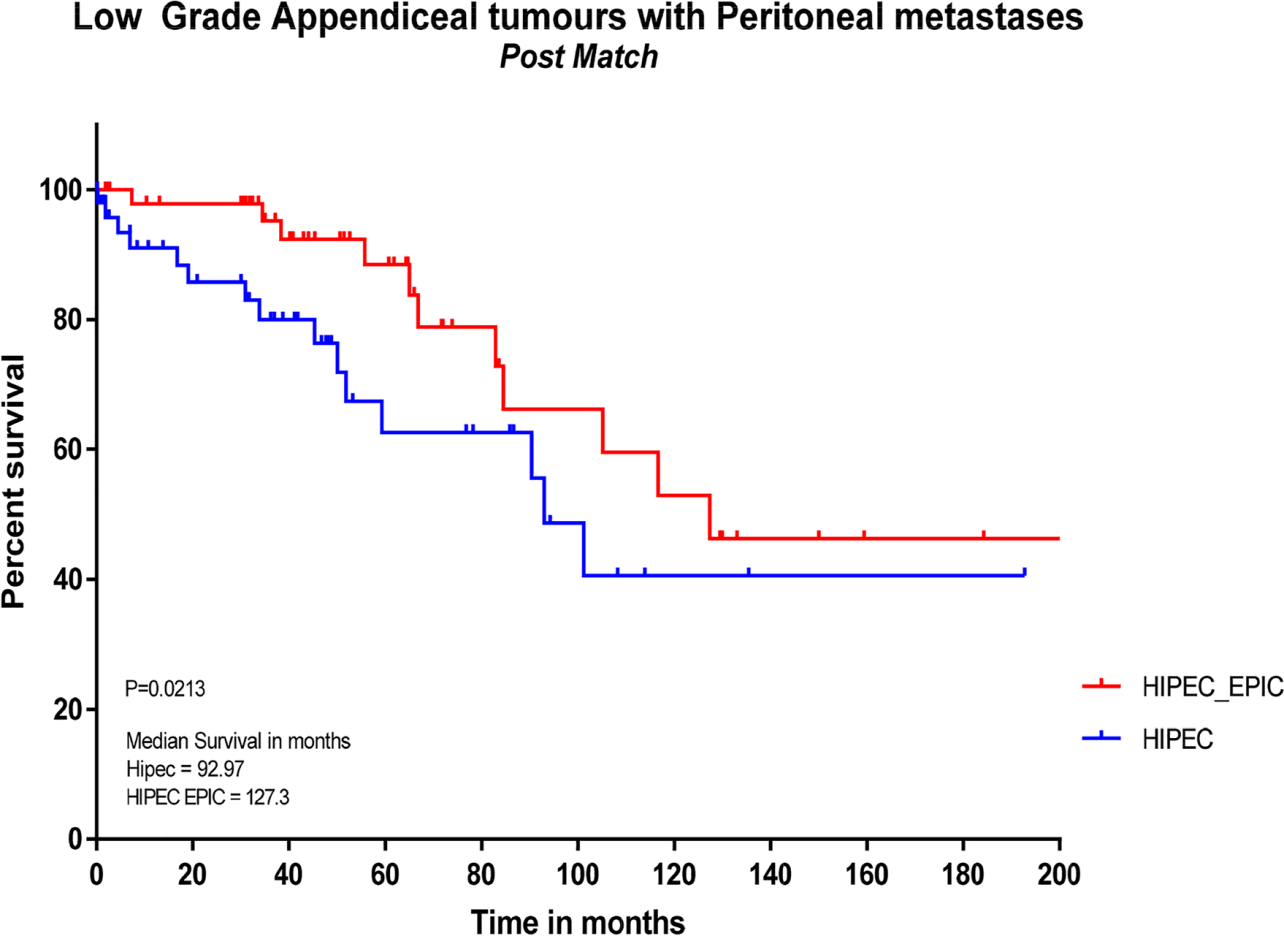
Kaplan-Meier curve of survival for patients treated with HIPEC or HIPEC + EPIC post-propensity matching. HIPEC, hyperthermic intraperitoneal chemotherapy; EPIC, early postoperative intraperitoneal chemotherapy.

Pre-and post-propensity matching Kaplan–Meier curves for survival are shown in Figures 1 and 2. Pre-propensity matching median survival (Figure 1) was 92.97 months in the HIPEC group, with HIPEC + EPIC not reaching a median with more than 50% surviving at time of analysis (p<0.001). Post-propensity matching median survival (Figure 2) was significantly different between the HIPEC and HIPEC + EPIC groups at 92.97 vs. 127.3 months, respectively (p=0.021).

## Discussion

This data shows a significant median survival benefit of 34.3 (127.3 vs. 93.0, p=0.0213) months with the addition of EPIC to HIPEC in a matched propensity study in patients with peritoneal spread of low grade appendiceal tumours. This was also associated with a median increase of 1.5 days total length of hospital stay (23.5 vs. 25.0, p=0.028).

There exists limited literature on the comparison of HIPEC to HIPEC + EPIC for LAMN. Sparks et al. showed no significant difference in overall survival for appendiceal cancer between HIPEC and HIPEC + EPIC, but combined both high and low grade in analysis and was likely underpowered to detect a difference (n=13 and 17 respectively, p=0.645) [17]. For HAMN, Lam et al. analysed survival for both colorectal and HAMN showing no significant survival benefit, but trend to better survival with 50% survival at 3 years compared to 46% for HIPEC + EPIC to HIPEC alone (p=0.72) [18]. As the included appendiceal tumours were high grade and different from our cohort, as well as making up only 25.8% (n=24) patients in their analysis, these are not translatable to our cohort. Chua et al. showed both appendiceal and colorectal cancers not to have survival benefit with additional EPIC to HIPEC, only showing disease free survival benefit with additional EPIC in cancers of colorectal origin [19], once again of differing pathology to our cohort. For appendiceal neoplasms with PMP alone; Soucisse et al. analysed the use of HIPEC with and without EPIC using propensity matching, identifying no benefit of EPIC even for LAMN, but noted those who had EPIC were from earlier in their centres learning curve and had higher mean CC score [20]. Huang et al. showed additional EPIC to be associated with improved OS for high-grade appendiceal cancer with a HR of 0.45 (p=0.004), but excluded those of low-grade [12]. Fung et al. in their series of HIPEC with and without EPIC for both low and high grade appendiceal cancer with PMP showed no survival difference, but also excluded patients undergoing high tumour debulking [21]. An additional study using an older version of this dataset in LAMN showed a 30.5% five-year overall survival benefit (93.0% for HIPEC+ EPIC vs. 64.5% for HIPEC alone, p<0.001), with median survival not reached [11]. Our study with its propensity score matching extends on this analysis and provides further evidence into the potential benefits of EPIC in LAMN with PMPS

Our major morbidity rates of 40.1% in the HIPEC+EPIC group was not different from the HIPEC group, pre-propensity matching (32.7%, p=0.492). Concern for higher morbidity with EPIC is common in the literature as a reason it fell out of favour, with trials from Tan et al. showing higher morbidity (58% vs. 25%, p=0.048), Glehen et al. showing higher rates of fistulas with a risk ratio of 1.7, p=0.032 and Lam et al. finding additional EPIC as an independent predictor of morbidity, with morbidity rates of 43.2% with EPIC vs. 19.6% of HIPEC alone [18, 22, 23]. Our morbidity rates in the HIPEC only group are higher than the reported rates in other series, but given our mean HIPEC PCI of 24.6, likely mirrors the extent of resection, with previous analyses of morbidity showing PCI>26 to be an independent predictor [24]. In comparison, Tan et al. with a morbidity rate of 25% in their HIPEC group reported a median PCI of 15 and 9 in their HIPEC cohorts, with likely a lower overall mean PCI [22]. It has been noted that recent work comparing HIPEC with HIPEC+EPIC appears to not show increased morbidity compared to older papers, possibly indicating better postoperative care [10].

Table 1 demonstrated differences in the baseline unmatched data of CC score and median ASA, which was not seen in the propensity matched cases (Table 2). This is likely a reflection of the strength of the study design, with propensity matching allowing these presumed covariates to be corrected for. The noted difference in age with those receiving EPIC being on average 6.2 years younger pre-matching also trended to correction, reducing to 4.1 years post-propensity matching. Age was not shown to impact survival at any stage of matching with a non-significant Pearson correlation of 0.018 and −0.05 pre and post matching respectively (Table 3). Whilst still a significant difference (p=0.044), the lack of impact on survival indicates this difference does not appear clinically significant in our cohort. Analyses from Glehen et al. had previously shown age not to affect survival until 65 years of age [23]. Similarly, CC score was also significantly lower in the HIPEC+EPIC group at baseline with evidence of impact on survival (Pearson correlation 0.231, p<0.001) (Tables 1 and 2), but this difference disappeared post-propensity matching with the matched group indicating that differing CC clearance between the groups did not affect survival. This likely explained by the median CC score being 0 in the matched groups, with less variance in scoring to allow for detection of CC impacting survival, which is a known factor.

Choice of HIPEC agent differed between the HIPEC alone and HIPEC+EPIC groups, with those receiving EPIC more likely to receive mitomycin C as opposed to oxaliplatin or cisplatin in both pre and post-propensity matching (Tables 1 and 2). This impacted survival pre-matching in multivariate analysis but propensity-matched survival was not affected by this choice, possibly highlighting the impact of covariables not corrected for in the unmatched influencing this. A large multicentre trial comparing oxaliplatin and mitomycin C in appendiceal tumours has previously shown no survival difference [25].

Finally, our total length of stay differed significantly in our post-propensity matched group, with the HIPEC + EPIC group experiencing a median longer length of stay when compared to HIPEC alone, at 25.0 vs. 23.5 days, p=0.028. ICU length of stay was not different. It is noted there are concerns of increasing ICU resources required for delivery of EPIC, but this was not shown in our data [10]. Shorter cycles of EPIC (1–2 days) have previously been shown to have no difference in survival benefits when compared to long course (3–5 days) EPIC [26]. The overall survival benefits likely outweigh the short median increase in length of stay for additional EPIC to HIPEC.

### Limitations

This is a single centre study examining the practices in one location, which may limit extrapolation to other centres, especially given the high-volume of our unit in this field. The retrospective nature limits conclusions when compared to a prospective randomised trial. Future directions could include a randomised controlled trial; similar to the current ICARuS trial being performed for colorectal primaries [27], but the rarity of this disease could limit enrolment. Morbidity was not analysed in the post-match group due to the propensity matching utilising median morbidity grade as a covariate in generation of matched samples for our primary outcome of survival. This study was not designed to examine this and a future analysis on types of post-operative complications and morbidity with different propensity-score matching is planned.

## Conclusions

In LAMN with PMP, the addition of EPIC to HIPEC with CRS significantly improves overall survival in propensity score matched cases and should be considered in select patients.
